# Moving a neodymium magnet promotes the migration of a magnetic tracer and increases the monitoring counts on the skin surface of sentinel lymph nodes in breast cancer

**DOI:** 10.1186/s12880-020-00459-2

**Published:** 2020-05-27

**Authors:** Masujiro Makita, Eriko Manabe, Tomoko Kurita, Hiroyuki Takei, Seigo Nakamura, Akihiro Kuwahata, Masaki Sekino, Moriaki Kusakabe, Yasuo Ohashi

**Affiliations:** 1 Department of Surgery, Breast Surgery Division, Nippon Medical School Musashikosugi Hospital, 1-396 Nakahara-ku, Kosugicho, Kawasaki, Kanagawa 211-8533 Japan; 2 Department of Breast Oncology, Nippon Medical School Hospital, Tokyo, Japan; 3 Breast center, Showa University Hospital, Tokyo, Japan; 4 Graduate School of Engineering, The University of Tokyo, Tokyo, Japan; 5 Matrix Cell Research Institute Inc., Ibaraki, Japan; 6 Graduate School of Agricultural and Life Sciences, The University of Tokyo, Tokyo, Japan; 7 Department of Integrated Science and Engineering for Sustainable Society, Chuo University, Tokyo, Japan

**Keywords:** Sentinel node biopsy, Breast cancer, Superparamagnetic iron oxide nanoparticles (SPIO), Neodymium magnet, Magnetometer

## Abstract

**Background:**

We suspected that moving a small neodymium magnet would promote migration of the magnetic tracer to the sentinel lymph node (SLN). Higher monitoring counts on the skin surface before making an incision help us detect SLNs easily and successfully. The present study evaluated the enhancement of the monitoring count on the skin surface in SLN detection based on the magnet movement in a sentinel lymph node biopsy (SNB) using superparamagnetic iron oxide (SPIO) nanoparticles.

**Methods:**

After induction of general anesthesia, superparamagnetic iron oxide nanoparticles were injected sub-dermally into the subareolar area or peritumorally. The neodymium magnet was moved over the skin from the injection site to the axilla to promote migration of the magnetic tracer without massage. A total of 62 patients were enrolled from February 2018 to November 2018: 13 cases were subjected to magnet movement 20 times (Group A), 8 were subjected to 1-min magnet movement (Group B), 26 were given a short (about 5 min) interval from injection to 1-min magnet movement (Group C), and 15 were given a long (about 25 min) interval before 1-min magnet movement using the magnetometer’s head (Group D). In all cases, an SNB was conducted using both the radioisotope (RI) and SPIO methods. The monitoring counts on the skin surface were measured by a handheld magnetometer and compared among the four groups. Changes in the monitoring count by the interval and magnet movement were evaluated.

**Results:**

The identification rates of the SPIO and RI methods were 100 and 95.2%, respectively. The mean monitoring counts of Group A, B, C, and D were 2.39 μT, 2.73 μT, 3.15 μT, and 3.92 μT, respectively (*p* < 0.0001; Kruskal-Wallis test). The monitoring counts were higher with longer magnet movement and with the insertion of an interval. Although there were no relationships between the monitoring count on the skin surface and clinicopathologic factors, magnet movement strongly influenced the monitoring count on the skin surface.

**Conclusion:**

Moving a small neodymium magnet is effective for promoting migration of a magnetic tracer and increasing monitoring counts on the skin surface.

**Trial registration:**

UMIN, UMIN000029475. Registered 9 October 2017

## Background

A sentinel lymph node biopsy (SNB) has been established as the standard method for staging clinically node-negative breast cancer [1, 2], and an SNB technique using superparamagnetic iron oxide (SPIO) nanoparticles and a handheld magnetometer has been reported [3]. While the radioisotope (RI) and dye-combined method has been thought to be the standard, an SNB using SPIO has been adopted because the RI method has disadvantages of radiation exposure [4], regulations regarding radioisotope management [5], and painful tracer injection [6, 7].

However, the SPIO method also has its own drawbacks, including the time needed to identify sentinel lymph nodes (SLNs) and the long-duration persistence of SPIO pigmentation. We suspected that that moving a small neodymium magnet would promote migration of the magnetic tracer to the SLN. Therefore, in a previous study, we waved a small neodymium magnet from the injection site to the axilla over the skin without massage after injection under general anesthesia during an SNB by SPIO. We found that this approach was useful for detecting SLNs, and the identification rate was extremely high with SPIO [8, 9].

One advantage of the RI or SPIO method is that the uptake is assessed as a quantitative monitoring count on the skin surface. Even in SNBs using indocyanine green (ICG) in gastric cancer, the fluorescence intensity in fluorescent nodes is reportedly evaluated using an ICG intensity imaging software program (the hyper eye medical system) [10]. Higher monitoring counts on the skin surface before making an incision can help us detect SLNs easily and successfully. In the RI method, the measured value can be trusted even if the count is 1, but the reliability of monitoring counts on the skin surface is reduced when the response of the magnetometer is weak, especially when the value is < 1 μT.

Changing the length of magnet movement or inserting an interval between the injection and magnet movement might be useful for increasing the count. We therefore evaluated the effect of moving the magnet on the monitoring count at the skin surface for SLN detection.

## Methods

The study was approved by the local ethics committees and was registered in the University hospital Medical Information Network (UMIN) Clinical Registry (UMIN000029475).

The subjects of the study were primary breast cancer patients diagnosed by a needle biopsy or fine-needle aspiration cytology who were ≥ 20 years old with no suspected axillary lymph node metastasis on imaging. We excluded cases with a history of breast and/or axillary surgery (such as after breast implant insertion), male breast cancer, and ipsilateral breast tumor recurrence after breast-conserving surgery. Patients who met the inclusion criteria were enrolled consecutively for this study. Written, informed consent was obtained from 69 patients from February to November 2018.

**Fig. 1:**
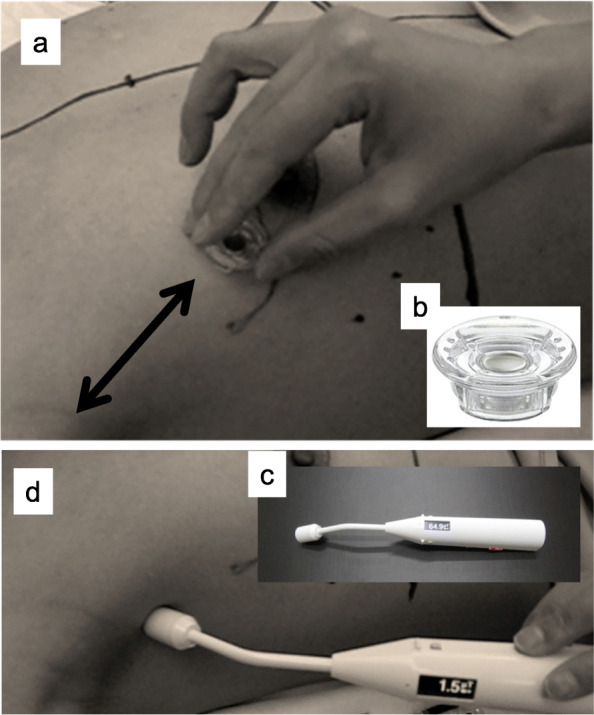
Magnet movement by a neodymium magnet. **a** A neodymium magnet was moved over the skin from the injection site to the axilla repeatedly to promote migration of the magnetic tracer without massage after the magnet tracer is injected. **b** A neodymium magnet (Neomag, KOKUYO Co., Ltd.). **c** The handheld magnetometer developed by Tokyo University. It contains a small neodymium magnet in its tip. **d** The monitoring count on the skin surface was measured by a handheld magnetometer

An SNB was conducted using both the RI and SPIO methods. Tc-99 m phytate was injected the day before the surgery at a dose of 74 MBq, and a dose of 37 MBq was given if patients were injected on the day of surgery. After induction of general anesthesia, 0.5 ml of ferucarbotran (Resovist® Inj.; FUJIFILM Toyama Chemical Co., Ltd., Tokyo, Japan) was injected sub-dermally into the subareolar area (total mastectomy case) or peritumorally (partial mastectomy case). A neodymium magnet (Neomag, KOKUYO Co., Ltd., Osaka, Japan) was moved over the skin 20 times from the injection site to the axilla to promote migration of the magnetic tracer without massage (Fig. 1). Drug injection and magnet movement were performed by two surgeons with no difference in height (MM, EM). Regarding the magnet movement procedure, the practitioner stood beside the affected breast of the patient and operated a wiper-like operation with the elbow as the axis at a distance of about 40 cm and a speed of about 1 sec for 1 round trip. The dye method of an SNB was not performed in addition to the RI method because of the omission of massage after performing injections in this study. Before the skin incision, the monitoring count on the skin surface was measured by a novel handheld magnetometer and confirmed twice. The magnetometer developed by Tokyo University contains a small neodymium magnet in its tip (Fig. 1) [11, 12]. After skin incision, if the removed node had a measurable RI count or a value exceeding 1 μT on the magnetometer, it was considered an SLN.

**Fig. 2:**
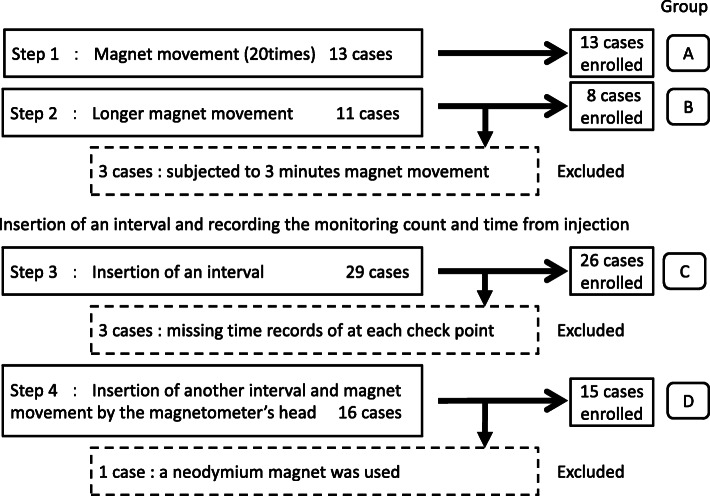
The consecutive steps and recruited cases in this study. Seven cases were excluded: three cases subjected to just three min of magnet movement, three cases with missing time records of at each check point, and 1 case in which a neodymium magnet instead of magnetometer’s head was used. We ultimately enrolled 62 out of the 69 cases

To determine how best to achieve a ≥ 1.5-μT count at the skin surface, the length of magnet movement was changed or an interval was inserted between the injection and magnet movement in this protocol. Several procedures were attempted in 69 cases consecutively: moving the magnet 20 times at the first step, changing the length of magnet movement at the second step, inserting an interval at the third step, and inserting another interval and magnet movement using the magnetometer’s head at the final step (Fig. 2). We proceeded to the next step after achieving higher counts than with the previous step. Based on these four steps, we set four groups of cases subjected to the same procedure (≥7 cases per group).

Seven cases were excluded: three cases subjected to just three minutes of magnet movement, three cases with missing time records at each check point, and one case in which a neodymium magnet instead of the magnetometer’s head was used. We therefore ultimately enrolled 62 of the 69 cases.

**Fig. 3:**
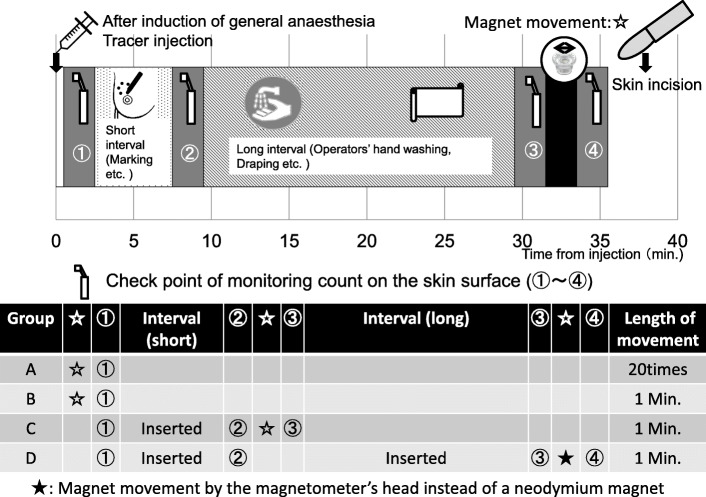
Measuring monitoring count and timing of the magnet movement of 4 cohorts. In Groups A and B, only the monitoring count after magnet movement was evaluated. In the 41 cases in Groups C and D, the monitoring count and time from injection were evaluated at certain check points, such as after injection, after an interval, before magnet movement, and after magnet movement. Changes in the monitoring count by interval and by magnet movement were evaluated

To compare the monitoring count by the length of the magnet movement or the interval from injection, 4 groups were analyzed: 13 cases were subjected to magnet movement 20 times (Group A), 8 were subjected to 1-min magnet movement (Group B), 26 were given a short (about 5 min) interval from injection to 1-min magnet movement (Group C), and 15 were given a long (about 25 min) interval before 1-min magnet movement using the magnetometer’s head (Group D). As a preparation for surgery before entering the cleanup operation, the time taken to confirm breast cancer using ultrasound and mark the resection area was set at a short interval. The interval from the operator’s hand washing to draping and just before the the start of skin incision was set at a long interval. In Groups A and B, only the monitoring count after the magnet movement was evaluated. In the 41 cases in Groups C and D, the monitoring count and time from injection were evaluated at certain check points, such as after injection, after the interval, before magnet movement, and after magnet movement (Fig. 3). The monitoring counts at the skin surface after magnet movement were compared among the four groups. Changes in the monitoring count by interval and by magnet movement were evaluated.

To compare the four groups, the χ2 test was used for variables presented as numbers of cases, and the Kruskal-Wallis test was used for those presented as the average value. Furthermore, when comparing the average values of the counts between the two groups, the Mann-Whitney test was used. Wilcoxon’s signed-rank test or Friedman’s test was used when comparing the average counts within the groups. The computer software program “Stat View for Windows version 4.54 “(Abacus Concepts, Inc., Berkley, CA, USA) was used for all analyses. Statistical associations were deemed significant at *P*-values < 0.05.

## Results

Table 1 shows the characteristics of the cases, and Table 2 shows the results of SNBs. The identification rates with the SPIO and RI methods were 100 and 95.2%, respectively. It took an average of 79.9 min from the injection of ferucarbotran to the removal of the SLN. Because we performed an SNB after making skin flap, the average time was slightly long. The lymph node retrieval rate was 3.0 nodes per patient overall, 2.0 nodes per patient with RI, and 2.9 nodes per patient with SPIO. There was no marked difference among the groups in the highest count of SLNs resected by the RI method (*p* = 0.2891). However, the mean values of the highest count of SLNs resected by SPIO differed significantly among the groups (*p* < 0.0001). Of the 183 SLNs removed, 125 (68.3%) were identified by RI, and 182 (99.5%) were identified by SPIO. Of the 19 SLNs that were histopathologically positive, 13 (68.4%) were identified by RI, and 19 (100%) were identified by SPIO. There were no cases with skin pigmentation after the operation, because the entire injected site was able to be resected during surgery.

**Table 1:** The characteristics of the cases

	Group	A	B	C	D	Total	χ2 test
	Cases	13	8	26	15	62	*p*-Value
Age (y)	(mean)	51.1	61.9	56.8	58.6	56.7	0.4072*
	≤39	3	2	3	2	10	0.4600
	40–49	4	0	7	3	14	
	50–59	1	1	7	2	11	
	60–69	4	1	5	4	14	
	≥70	1	4	4	4	13	
Body mass index (kg/m^2^)	(mean)	22.2	25.0	22.3	23.5	22.9	0.6837*
	< 25	10	5	21	11	47	0.7711
	≥25	3	3	5	4	15	
Tumor location	Lateral/Central	9	8	19	9	45	0.2316
	Medial	4	0	7	6	17	
T Classification	Tis	3	1	4	4	12	0.8833
	T1	9	6	19	8	42	
	T2	1	1	2	3	7	
	T4	0	0	1	0	1	
Assessment preoperative axillary lymph node	US/MRI/CT	12	8	26	15	61	0.2803
	Confirmed by FNA	1	0	0	0	1	
Surgery	Partial mastectomy	9	6	16	8	39	0.7210
	Mastectomy	4	2	10	7	23	
Histology	DCIS	3	0	5	5	13	0.3752
	IDC	9	5	16	6	36	
	Others	1	3	5	4	13	

*: Kruskal-Wallis test

**Table 2:** The results of SNB

	Group	Total	A	B	C	D	Kruskal-Wallis test
	Cases	62	13	8	26	15	*p*-Value
Time from injection to removal of the SLN	(mean)	79.9	71.7	88.8	83.1	76.3	0.1048
	(range)	45 ~ 120	45 ~ 95	70 ~ 120	55 ~ 110	50 ~ 100	
SLN detection by RI method	Success	59	13	6	25	15	0.0361*
	Failure	3	0	2	1	0	
Lymph node retrieval rate (nodes)	Overall	3.0	3.0	3.3	2.5	3.5	0.5524
	RI method	2.0	2.3	1.5	1.8	2.5	0.7357
	SPIO method	2.9	3.0	3.3	2.5	3.4	0.5524
The highest count of resected SLN (RI)	(mean)	839.4	350.8	231.3	538.5	2108.7	0.2891
	(range)	0 ~ 21,500	70 ~ 1100	0 ~ 800	0 ~ 4000	40 ~ 21,500	
The highest count of resected SLN (SPIO)	(mean)	3.03	2.18	2.81	2.96	4.02	< 0.0001
	(range)	1.5 ~ 11.0	1.5 ~ 3.5	2.0 ~ 4.5	2.0 ~ 4.0	2.5 ~ 11.0	
Metastasis of SLN	None	50	12	6	18	14	0.3889*
	Micrometastasis	2	0	0	2	0	
	Macrometastasis	10	1	2	6	1	

*: χ2 test

Table 3 shows the results of the comparison among the four groups. The mean monitoring counts of Groups A, B, C, and D were 2.39 μT, 2.73 μT, 3.15 μT, and 3.92 μT, respectively (*p* < 0.0001; Kruskal-Wallis test). The monitoring counts were higher with longer magnet movement and with the insertion of an interval.

**Table 3:** The results of the comparison among the 4 groups

Group	Magnet movement		Cases	Monitoring count on the skin surface (μT)			
	Length	Timing		Mean*	Range	Mann-Whitney test	
A	20 times	After injection	13	2.39	1.5 ~ 3.5		*p* = 0.0607
B	1 min		8	2.73	2.5 ~ 3.0	*p* = 0.0130	
C		After an interval (short)	26	3.15	2.0 ~ 4.8		*p* = 0.0029
D		After an interval (long)	15	3.92	2.8 ~ 6.0		
					*:*p*<0.0001	(Kruskal-Wallis test)	

**Fig. 4:**
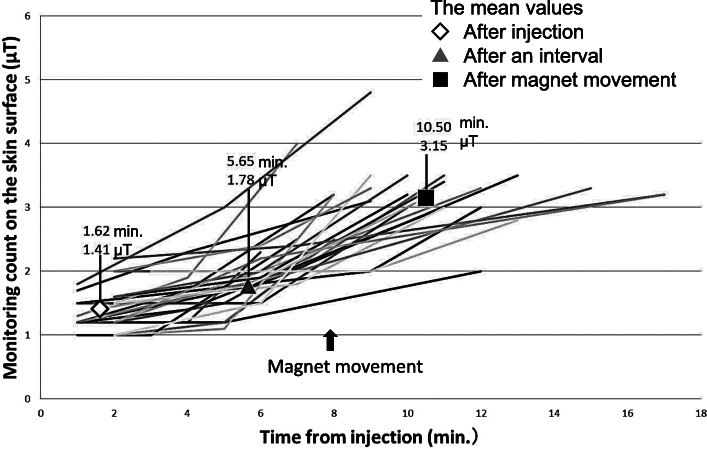
Relationship between the time from injection and monitoring count in Group C. Sequential lines indicate each evaluated cases. Symbols show the mean values at check points, such as after injection, after an interval, and after magnet movement

**Fig. 5:**
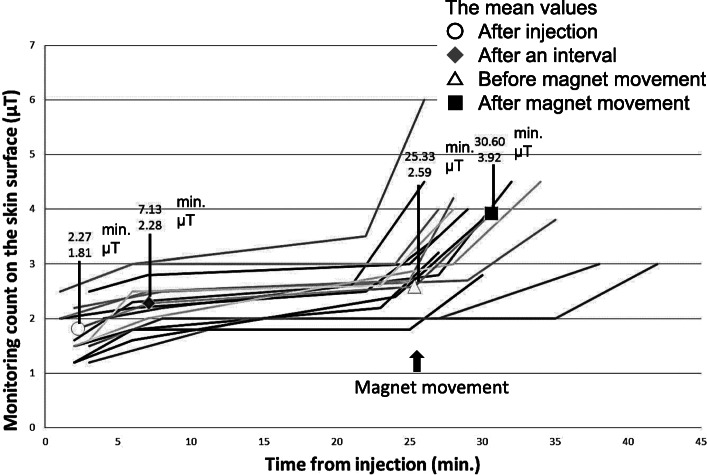
Relationship between time from injection and the monitoring count in Group D. Sequential lines indicate each evaluated cases. Symbols show the mean values at check points, such as after injection, after an interval, before magnet movement, and after magnet movement

The relationship between the time from injection and monitoring count in Groups C and D is shown as a scattergram in Figs. 4 and 5, respectively. Sequential lines indicate each evaluated case. Symbols show the mean values at check points, such as after injection, after an interval, before magnet movement, and after magnet movement. Although there were some cases in which the monitoring counts were ≥ 1.5 μT after injection, the monitoring counts increased after magnet movement in all cases.

**Fig. 6:**
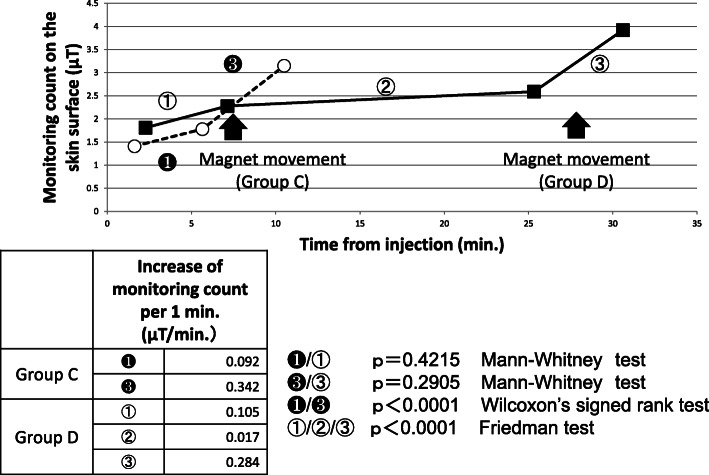
Sequences of the mean values at each check point and the monitoring count gains per minute. The large arrow shows the timing of magnet movement in each group. The monitoring count increases were significantly greater during magnet movement than after injection or during an interval

Sequences of the mean values at each check point in Groups C and D are shown in Fig. 6. The monitoring count gains per minute at each time point are also shown. At the same time points, there were no marked differences between Groups C and D. However, in the same group, the monitoring count increases were significantly greater during magnet movement than after injection or during an interval. The monitoring counts increased gradually with time, but they showed a greater increase during magnet movements for a short period of time than without such magnetic movements.

Although magnet movement strongly influenced the monitoring count at the skin surface, there were no remarkable relationships between the monitoring count at the skin surface and clinicopathologic factors (Table 4).

**Table 4:** Monitoring count on the skin surface and clinicopathologic factors

Factor		Cases	Monitoring count on the skin surface (μT)		
			Mean	Range	*P* value
Age (y)	≤39	10	3.36	2.0 ~ 6.0	*p* = 0.4423, *2
	40–49	14	3.33	2.5 ~ 4.5	
	50–59	11	3.04	2.0 ~ 3.8	
	60–69	14	2.79	1.5 ~ 4.0	
	≥70	13	3.13	2.0 ~ 4.5	
Body mass index (kg/m^2^)	< 25	47	3.15	2.0 ~ 6.0	0.8297, *1
	≥25	15	3.04	1.5 ~ 4.0	
Tumor location	Lateral/Central	45	3.11	1.5 ~ 4.8	0.7268, *1
	Medial	17	3.14	2.0 ~ 6.0	
T Classification	Tis	12	3.18	1.5 ~ 4.8	0.3634, *2
	T1	42	3.06	2.0 ~ 6.0	
	T2/T4	8	3.35	2.5 ~ 4.0	
Surgery	Partial mastectomy	39	3.01	2.0 ~ 4.5	0.2466, *1
	Mastectomy	23	3.30	1.5 ~ 6.0	
Histology	DCIS	13	3.12	1.5 ~ 4.0	0.7149, *2
	IDC	36	3.12	2.0 ~ 6.0	
	Others	13	3.12	2.5 ~ 4.5	
SLN detection by RI method	Success	59	3.13	1.5 ~ 6.0	0.7413, *1
	Failure	3	2.93	2.5 ~ 3.3	
Metastasis of SLN	None	50	3.18	1.5 ~ 6.0	0.1867, *2
	Micrometastasis	2	2.40	2.3 ~ 2.5	
	Macrometastasis	10	2.95	2.0 ~ 4.0	
		*1: Mann-Whitney test, *2: Kruskal-Wallis test			

## Discussion

An SNB has been established as the standard method for staging clinically node-negative breast cancer [1, 2]. The benefits of an SNB performed by SPIO include the lack of radiation exposure, the fact that it can be performed at any hospital regardless of the presence of a radioisotope department. Indeed, with the SPIO method, the location of SLNs or non-palpable breast tumors can also be identified by a detector before a skin incision is made similar to the RI method [3, 13–17].

Thill reported that an SNB using SentiMag® and Sienna+® (Endomagnetics, Inc., Austin, TX, USA) was useful in a multicenter study using magnetic techniques to detect SLNs for breast cancer [15]. An SNB was performed using ferucarbotran (Resovist® Inj.; FUJIFILM Toyama Chemical Co., Ltd.) and a novel handheld magnetometer developed by Tokyo University in the present study. This method has drawbacks, including the time needed to identify SLNs and the long-duration persistence of SPIO pigmentation. We therefore performed magnet movement using a small neodymium magnet to promote the migration of the magnet tracer in a previous study [9]. That study involved 69 patients evaluated from March 2017 to January 2018. After the induction of general anesthesia, 0.3 ml of ferucarbotran was injected into the subareolar area or peritumorally. The identification rate was 98.6% (68/69) with RI and 100% (69/69) with SPIO. The identification rate using the SPIO method with magnet movement was estimated to be better than 95% (90% confidence interval: 95.75–100%). In contrast, the identification rates of RI methods were slightly low (95.2%) in the present study. However, in that previous study, the identification rate was 98.6% (68/69, 90% confidence interval: 93.3–99.9%) with RI in our hospital, and the value of 95.2% falls within that confidence interval.

When using the RI method, it is easy to detect SLNs because the RI probe can detect the radiation beam from SLNs. However, it is slightly difficult to detect SLNs by SPIO, as the magnetometer must seek out a small tracer collection point. The purpose of the present protocol was to determine how best to obtain a higher count at the skin surface. To this end, the usefulness of magnet movement was evaluated from the perspective of the monitoring count at the skin surface. After increasing the dose of ferucarbotran from 0.3 ml (previous study) to 0.5 ml (Group A in the present study) and moving the magnet 20 times, the mean monitoring count increased significantly from 1.37 μT to 2.39 μT (*p* < 0.0001, Mann-Whitney test), and the identification rate of SPIO was 100% [9]. None of the patients showed any pigmentation despite the dose escalation. In subsequent steps, the length of magnet movement was changed, or an interval was inserted between injection and magnet movement to obtain a higher count at the skin surface. The monitoring counts increased with longer magnet movement as well as with insertion of an interval. The monitoring counts of the resected SLNs were comparable to those at the skin surface. These increased monitoring counts at the skin surface helped us detect SLNs easily and successfully. Ultimately, 1-min magnet movement with the magnetometer’s head approximately 30 min after tracer injection was found to be the best procedure for obtaining a higher monitoring count.

A small neodymium magnet was contained in the tip of the magnetometer developed by Tokyo University, and the magnetic force of this magnet was about five times as strong as the neodymium magnet Neomag. Several factors, including obesity and age [18, 19], have been reported to affect the outcome of an SNB, but no relationships were noted between the SPIO method and these clinicopathologic factors in the present study. Furthermore, of the 19 SLNs that were histopathologically positive, 13 (68.4%) were identified by RI, and 19 (100%) were identified by SPIO. There was actually one case in which one SLN that could not be identified by the RI method but that could be identified by the SPIO method was positive for metastasis. Movement of a small neodymium magnet to promote migration of the magnetic tracer is thus considered to be a promising method to employ during an SNB using SPIO, based on the identification rate, enhanced monitoring count, and the precise and optimal detection of SLNs.

The principles of an SNB are that injected small molecules pass through lymphatic vessels from the injected site and leach into the nodes through the lymphatic flow. Thus, the outcome of an SNB is affected by several factors, including tracer infiltration into the lymphatic vessels, the flow of lymph, and lodging in the nodes. To improve tracer infiltration into the lymphatic vessels, a longer period from injection to detection [19, 20] and massage after injection [21] have been applied previously. While these approaches did result in a small amount of tracer leaching into the nodes, the majority of the tracer failed to do so, instead spreading into the surrounding breast tissue. In such cases, skin pigmentation can occur clinically if the tracer is colored [20]. The lymph flow is affected by not only patient factors, including age and obesity, but also by tracer factors, such as the particle size [22]. While ferucarbotran is small enough to flow smoothly, it has also been found to be taken into neutrophils through phagocytosis and lodged in the lymph nodes. Inducing movement using a neodymium magnet is useful and expected to localize the tracer to SLNs smoothly and certainly when performing SNBs by SPIO.

Because a SNB is an intraoperative examination performed under general anesthesia, the patients could not be imaged twice (once with the magnet movement and once without it) in order to ensure that the same lymph nodes were marked. In addition, the detection of SLNs is based on the priority of the lymph nodes that receive the lymph flow, so the number of SLNs may differ depending on the timing of observation, and the same result may not be obtained even when performing imaging evaluations, such as computed tomography.

From the perspective of priority, the present results suggest that the RI and SPIO methods have similar priorities. This is because all but one lymph node detected by the RI method were detected by the SPIO method, and the lymph node detected only by the RI method also had a count of 0.8 μT by the SPIO method. In addition, there is some concern in the present study whether or not magnet movement produces a non-physiological lymphatic flow. However, no infiltration of the magnetic tracer into the skin was detected, and the infiltration of the magnetic tracer was not concentrated in the direction of the magnet movement. Furthermore, it was observed with the naked eye that the magnetic tracer even reached the margins of the resected lymph node, similar to the dye method, and it could also be histologically confirmed in the subcapsular sinus of the lymph node. We therefore believe that magnet movement did not create new anatomical lymph vessels but instead simply changed the speed of the physiological flow in existing lymph vessels.

Several limitations associated with the present study warrant mention. The number of patients in each group was not set in advance because the method changed while devising new ways to increase the count at the skin surface. Furthermore, because of the consecutive nature of the enrollment, background factors, such as age and obesity, could not be organized. An SNB is an intraoperative examination performed under general anesthesia that ends with the removal of lymph nodes and thus cannot be repeated in the same patient. Finally, this was not a randomized controlled trial.

The present findings suggest that, when performing an SNB by the SPIO method, the addition of magnet movement facilitated the identification of SLNs before surgery. This approach was also able to be performed in a relatively short time after the introduction of general anesthesia in a hospital without a radiation-controlled area. Patients can also avoid pain due to the injection or radiation exposure that must be endured with the RI method.

The RI method is the standard for SNB, and the amount of radiotracer may be minimal. However, it has been reported that the operator reaches the maximum allowable exposure level for 1 year (i.e. 1 mSv) after 333 operations [4].

The magnetic movement accelerated the speed of the magnetic tracer flow in the lymph vessels and increased the accumulation in the lymph nodes. This approach may also be used as a new drug delivery system for increasing the concentration of specific drugs in specific organs.

## Conclusion

Magnet movement using a small neodymium magnet from the injection site to the axilla over the skin without massage after injection under general anesthesia was performed in order to promote migration of a magnetic tracer in an SNB by SPIO. The movement was evaluated based on the monitoring count at the skin surface, and this approach was found to be useful for promoting the migration of the magnetic tracer and thereby obtaining higher monitoring counts at the skin surface. Magnet movement during an SNB by SPIO can be performed easily and certainly during surgery without causing pigmentation.

## Data Availability

The data used and/or analyzed during the current study are available from the corresponding author on reasonable request.

## Abbreviations

SLN: Sentinel lymph node
SNB: Sentinel lymph node biopsy
SPIO: Superparamagnetic iron oxide
RI: Radioisotope
ICG: Indocyanine green
UMIN: The University hospital Medical Information Network
